# Comprehensive Analysis of Ferroptosis- and Immune-Related Signatures to Improve the Prognosis and Diagnosis of Kidney Renal Clear Cell Carcinoma

**DOI:** 10.3389/fimmu.2022.851312

**Published:** 2022-05-10

**Authors:** Xiao-Liang Xing, Yan Liu, Jiheng Liu, Huanfa Zhou, Huirong Zhang, Qi Zuo, Ping Bu, Tong Duan, Yan Zhou, Zhiquan Xiao

**Affiliations:** ^1^Department of General Medicine, University of South China affiliated Changsha Central Hospital, Changsha, China; ^2^School of Public Health and Laboratory Medicine, Hunan University of Medicine, Huaihua, China; ^3^Department of Emergency, First Hospital of Changsha, Changsha, China

**Keywords:** KIRC, methylation, ferroptosis, immune, prognosis, diagnosis

## Abstract

**Background:**

Almost 40% of patients with kidney renal clear cell carcinoma (KIRC) with advanced cancers eventually develop to metastases, and their 5-year survival rates are approximately 10%. Aberrant DNA methylations are significantly associated with the development of KIRC. The aim of our present study was to identify suitable ferroptosis- and immune-related (FI) biomarkers correlated with aberrant methylations to improve the prognosis and diagnosis of KIRC.

**Methods:**

ChAMP and DESeq2 in R (3.6.2) were used to screen the differentially expressed methylation probes and differentially expressed genes, respectively. Univariate and multivariate Cox regression were used to identify the overall survival (OS)–related biomarkers.

**Results:**

We finally identified five FI biomarkers (*CCR4*, *CMTM3*, *IFITM1*, *MX2*, and *NR3C2*) that were independently correlated with the OS of KIRC. The area under the curve value of the receiver operating characteristic value of prognosis model was 0.74, 0.68, and 0.72 in the training, validation, and entire cohorts, respectively. The sensitivity and specificity of the diagnosis model were 0.8698 and 0.9722, respectively. In addition, the prognosis model was also significantly correlated with several immune cells and factors.

**Conclusion:**

Our present study suggested that these five FI-DEGs (*CCR4*, *CMTM3*, *IFITM1*, *MX2*, and *NR3C2*) could be used as prognosis and diagnosis biomarkers for patients with KIRC, but further cross-validation clinical studies are still needed to confirm them.

## Introduction

Kidney cancer is the second most common malignancies in the urinary system, which accounts for 2.2% of all new cancer cases, with over 430,000 new cases and 1.8% of all cancer related death, with almost 180,000 deaths in 2020 globally (1). Kidney renal clear cell carcinoma (KIRC) is the most common subtype, which accounts for 75% of kidney cell carcinomas (2, 3). Surgery is the primary treatment for KIRC (4, 5). However, almost 40% of patients with KIRC with advanced cancers eventually develop to metastases despite of early surgical treatment carried out (6, 7). Patients with KIRC have poor prognosis and high mortality rates, and their 5-year survival rates are approximately 10% (3, 8). Therefore, it is very necessary to identify potential prognosis biomarkers for the diagnosis and prognosis of KIRC.

Epigenetics was originally coined by Conrad Waddington, which plays a critical role in the regulation of DNA-based processes (9). Consequently, abnormal expression patterns or genomic alterations caused by abnormal epigenetic modifications may induce and maintenance various cancers (9). Accumulating pieces of evidence indicate that epigenetic alterations are the early events of cancerigenesis (9–11). For example, several key cancerigenesis related pathways could be regulated by epigenetic, such as Wnt/β-catenin signaling pathway, Hedgehog signaling pathway, and Notch signaling pathway (12–14). DNA methylations as the typical epigenetic manners are indeed involved in procession of many cancer stem cells, such as leukemic, lung and colon stem cells (15–17). In addition, previous studies have also demonstrated that aberrant DNA methylations are significantly correlated with the procession of KIRC (18, 19).

Numerous studies demonstrated that ferrptosis and immune can regulate each other and participate in the progression of several cancers (20–25). Regulation of ferroptosis and immune are currently considered to be novel therapeutic targets for the cancers (24, 26–29). The Cancer Genome Atlas (TCGA) is an open access database. In the present study, we downloaded 484 methylation data, 602 RNA sequencing (RNA-seq) data and the corresponding clinical information from TCGA and aimed to identify suitable ferroptosis- and immune-related (FI) biomarkers correlated with aberrant methylations to improve the prognosis and diagnosis of KIRC.

## Materials and Methods

### Data Source and Data Processing

The data used in present study were obtained from an open access database TCGA, including 484 samples (160 controls vs. 324 cancers) of methylation data, 602 samples (72 controls vs. 530 cancers) of RNA-seq data, and the corresponding clinical information. The recognized FI-related genes were obtained from the FerrDb and ImmPort, respectively. We used ChAMP and DESeq2 in R (3.6.2) to screen the differentially expressed methylatyion probes (DMPs) as the criteria padj < 0.05 and |logFC| ≥ 0.2 and differentially expressed genes (DEGs) as the criteria padj < 0.05 |logFC| ≥ 0.5 and base mean ≥ 100, respectively. Pearson correlation analyses were used to determine the relationship of DEGs and their corresponding DMPs as the criteria R ≤ −0.3. To construct a prognostic model and verify it, 530 patients with KIRC were randomly separated into training (n = 354) and validation (n = 176) cohorts (**Table 1**).

**Table 1:** Clinical features of patients in the training cohort and validation cohort.

Variables		Training Cohort (n = 354)		Validation Cohort (n = 176)	
		No.	%	No.	%
**Age**	≤65	229	64.69	119	67.61
	>65	125	35.31	57	32.39
**Stage**	I	183	51.69	82	46.59
	II	34	9.60	23	13.07
	III	84	23.73	39	22.16
	IV	52	14.69	30	17.05
	X	1	0.28	2	1.14
**T**	T1	187	52.82	84	47.73
	T2	40	11.30	29	16.48
	T3	120	33.90	59	33.52
	T4	7	1.98	4	2.27
**N**	N0	165	46.61	74	42.05
	N1	9	2.54	7	3.98
	NX	180	50.85	95	53.98
**M**	M0	283	79.94	137	77.84
	M1	49	13.84	29	16.48
	MX	22	6.21	10	5.68
**Gender**	Female	131	37.01	55	31.25
	Male	223	62.99	121	68.75

### Construction of Prognosis and Diagnostic Model

We divided the patients with KIRC into low-expression cohort and high-expression cohort by the median expression value. The univariate Cox regression and multivariate Cox regression in R (3.6.2) were used to identify the candidate biomarkers. After multivariate Cox hazards regression, we constructed the prognostic model according to the previous reports (30, 31). Risk score = (−0.9653) × Exp_(CCR4)_ + (−0.7026) × Exp_(CMTM3)_ + (0.8370) × Exp_(IFITM1)_ + (0.8450) × Exp_(MX2)_ + (−0.7717) × Exp_(NR3C2)_

Patients with KIRC were divided into low-risk cohort and high-risk cohort depends on the optimal cutoff value (Youden Index).

After a stepwise logistic regression analyses, the diagnostic model was constructed as follows: LOGIT score = 0.7998 + (0.1034)*Exp_(CMTM3)_ + (−0.1590)*Exp_(NR3C2)_ + (0.0465)*Exp_(MX2)_ + (0.0737)*Exp_(CCR4)_ + (0.0966)*Exp_(IFITM1)_

### Enrichment Analyses and Principal Component Analyses

David 6.8 was used to carry out Gene Ontology (GO) and Kyoto Encyclopedia of Genes and Genomes (KEGG) enrichment analyses with the default parameter (https://david.ncifcrf.gov/). A principal component analyses (PCAs) in R (3.6.2) was used to reduce the dimensions and to visualize the distribution of the patients with KIRC.

### Statistical Methods

A repeated-measure ANOVA followed by Bonferroni *post hoc* tests or unpaired two-tail Student’s t-test was used as indicated.

## Results

### Identification of Candidate Prognostic Biomarkers

**Figure 1:**
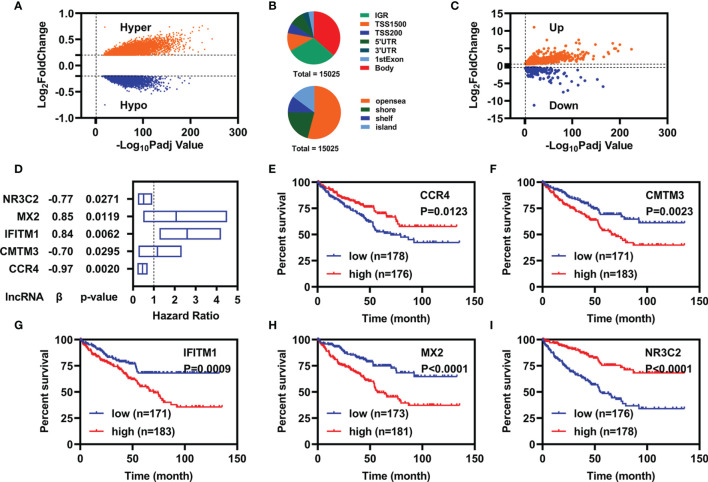
Identification of candidate prognostic biomarkers. **(A)** Volcano plot of DMPs between normal and patients with KIRC. **(B)** Distribution of DMPs by considering the CpG content (up) and the neighboring context (down). **(C)** Volcano plot of FI-DEGs between normal and patients with KIRC. **(D)** Multivariate Cox regression analyses illustrated five FI-DEGs independently correlated with OS. **(E–I)** Overall survival status for these five FI-DEGs.

We downloaded methylation data of 484 samples (160 controls vs. 324 cancers) from the TCGA database and obtained 15,025 DMPs by ChAMP. Of which, 9,294 were hypermethylated DMPs and 5,731 were hypomethylated DMPs (**Figure 1A**). The distributions of those 15,025 DMPs were showed in **Figure 1B** by considering the CpG content and the neighboring context.

Similarly, we also downloaded RNA-seq data of 602 samples (72 controls vs. 530 cancers) from TCGA database and obtained 7,500 DEGs (4,515 were upregulated DEGs and 2,985 were downregulated DEGs) through DESeq2 (**Supplementary Figure 1**). Of which, there were 784 DEGs were FI-DEGs (**Figure 1C**). Then, we introduced Pearson correlation analyses for those 15,025 DMPs and their corresponding FI-DEGs and found that there were 138 FI-DEGs correlated with 256 DMPs (**Supplementary Table 1**).

To obtain suitable FI-DEGs as biomarkers, we firstly performed the univariate Cox regression analyses for those 138 FI-DEGs correlated with DMPs and found 61 FI-DEGs were correlated with the overall survival (OS) of patients with KIRC in the training cohort. We then performed the multivariate Cox regression analyses for those 61 FI-DEGs and found that five of 61 FI-DEGs (*CCR4*, *CMTM3*, *IFITM1*, *MX2*, and *NR3C2*) were independently correlated with the OS of patients with KIRC (**Figure 1D**). Kaplan–Meier (KM) curve showed patients with KIRC with high expression of CCR4 and NR3C2 displayed better OS, whereas patients with high expression of CMTM3, IFITM1, and MX2 displayed worse OS (**Figures 1E–I**).

### Specific Prognostic Model Construction

After multivariate Cox regression analyses, we constructed a specific prognostic model using those five FI-DEGs. Depending on the Youden Index as the optimal cutoff value (**Supplementary Figure 2**), we regrouped the patients with KIRC into low-risk cohort and high-risk cohort. The expressions of these five FI-DEGs between patients with KIRC with the low-risk cohort and high-risk cohort were displayed in **Supplementary Figures 3A–C**. The correlations of these five FI-DEGs with the risk value were displayed in **Supplementary Figures 3D–F**. CCR4, IFITM1, and MX2 were significantly correlated with the prognostic model (**Supplementary Figures 3D–F**).

**Figure 2:**
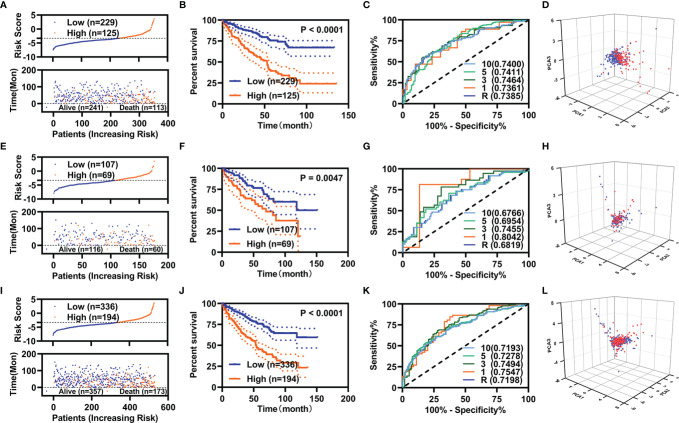
Specific prognostic model constructions. **(A–D)** For the training cohort. **(A)** Risk value (up) and survival status (down), **(B)** KM curve, **(C)** ROC curve, and **(D)** PCA analyses. **(E–H)** For the validation cohort. **(E)** Risk value (up) and survival status (down), **(F)** KM curve, **(G)** ROC curve, and **(H)** PCA analyses. **(I–L)** For the entire cohort. **(I)** Risk value (up) and survival status (down), **(J)** KM curve, **(K)** ROC curve, and **(L)** PCA analyses.

We constructed prognostic models using these five FI-DEGs in the training, validation, and entire cohort. In the training cohort, the risk score (up) and survival status (down) for each patients with KIRC were displayed in **Figure 2A**. Patients with KIRC with low-risk value had longer survival time [**Figure 2A** (down)]. KM curve showed patients with KIRC with low-risk value displayed better OS (**Figure 2B**). The time dependent area under the curve (AUC) value of receiver operating characteristic (ROC) of the prognostic model was displayed in **Figure 2C**. All AUC values were over 0.70. The patients with high-risk value could well be distinguished from the whole patients as measured by the PCA analyses (**Figure 2D**). To determine whether these five biomarkers were feasible, we conducted validation studies in validation cohort and entire cohort. The similar results were displayed in **Figures 2E-L**. In all three cohorts, the 5-year AUC value of the prognostic model reached 0.70 (**Figures 2C, G, K**). These results indicated the prognostic model constructed using those five FI-DEGs could well predict the outcome of patients with KIRC.

### Clinical Evaluation of the Prognostic Model

**Figure 3:**
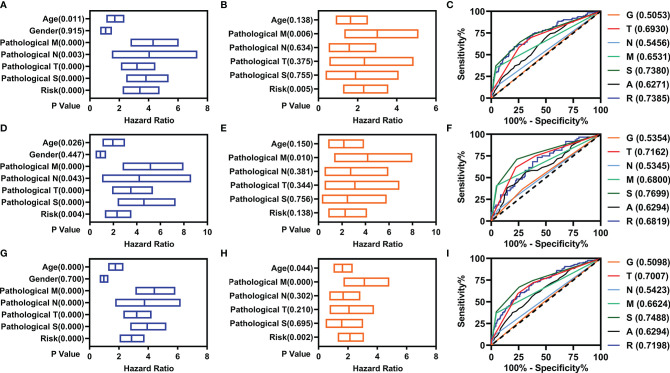
Independent prognostic factors analyses. **(A–C)** For the training cohort: **(A)** Univariate Cox regression analyses, **(B)** multivariate Cox regression analyses, and **(C)** ROC curve. **(D–F)** For the validation cohort: **(D)** Univariate Cox regression analyses, **(E)** multivariate Cox regression analyses, and **(F)** ROC curve. **(G–I)** For the entire cohort: **(G)** Univariate Cox regression analyses, **(H)** multivariate Cox regression analyses, and **(I)** ROC curve.

To know the role of the prognostic model in the prediction, we performed univariate and multivariate Cox regression analyses for the prognostic model and the variant clinical features. In the training cohort, the age, pathological TNM, pathologic stage, and the prognostic model were correlated with the OS as measured by univariate Cox regression analyses (**Figure 3A**). The pathological M and prognostic model were independently correlated with the OS as measured by multivariate Cox regression analyses (**Figure 3B**). In addition, the AUC value of prognostic model was high than that of the pathological M (**Figure 3C**).

In the validation cohort, the age, pathological TNM, pathologic stage, and the prognostic model were correlated with the OS as measured by univariate Cox regression analyses (**Figure 3D**). The pathological M was independently correlated with the OS as measured by multivariate Cox regression analyses (**Figure 3E**). The AUC value of prognostic model was comparable with that of the pathological M (**Figure 3F**).

In the entire cohort, the age, pathological TNM, pathologic stage, and the prognostic model were correlated with the OS as measured by univariate Cox regression analyses (**Figure 3G**). The age, pathological M, and prognostic model were independently correlated with the OS as measured by multivariate Cox regression analyses (**Figure 3H**). The AUC value of prognostic model was higher than that of the age and pathological M (**Figure 3I**).

**Figure 4:**
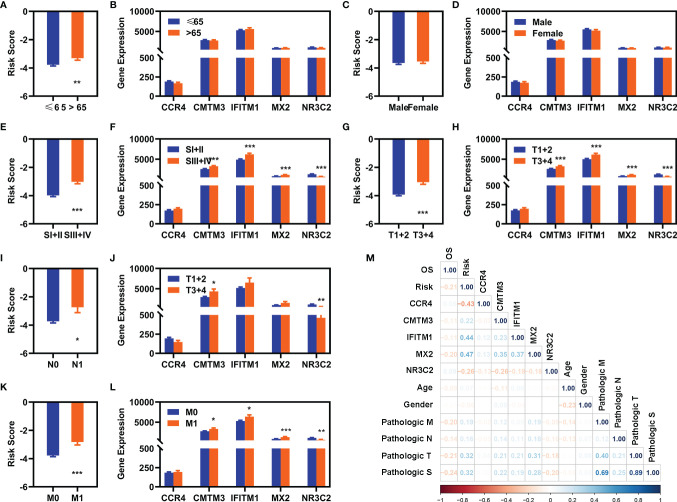
Correlation analyses with clinical features. **(A**, **C**, **E**, **G**, **I**, **K)** Differentially expression analyses of risk value with clinical different features in the entire cohort [**(A)** age, **(C)** gender, **(E)** pathological S, **(G)** pathological T, **(I)** pathological **(N)**, and **(K)** pathological **(M)**]. **(B)**, **(D)**, **(F)**, **(H)**, **(J)**, **(L)** Differentially expression of these five FI-DEGs with clinical features in the entire cohort [**(A)** age, **(C)** gender, **(E)** pathological S, **(G)** pathological T, **(I)** pathological **(N)**, and **(K)** pathological **(M)**]. **(M)** Correlation analyses of these five FI-DEGs and risk value with the clinical features in the entire cohort. N (≤65) = 348, n (>65) = 182. N (Male) = 344, N (Female) = 186. N (SI + II) = 322, N (III + IV) = 205. N (T1 + 2) = 340, N (T3 + 4) = 190. N (N0) = 239, N (N1) =16. N (M0) = 420, N (M1) = 78. *p < 0.05, **p < 0.01, ***p < 0.001.

We also explored the relationship of the expression of these five FR-DEGs and the risk value with the clinical features. The results were displayed in **Figure 4**.

### Correlation Analyses of the Prognostic Model With the Immunity

**Figure 5:**
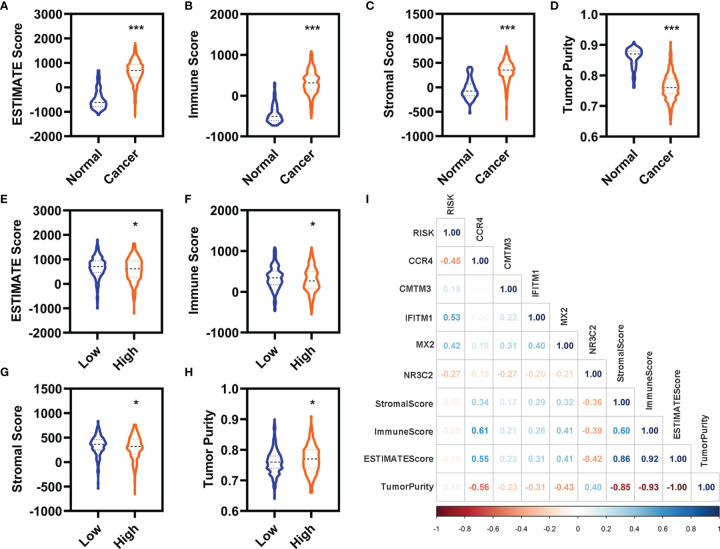
Correlation analyses with the immune. **(A–D)** Differentially expressed analyses for the ESTIMATE **(A)**, immune **(B)**, stromal **(C)**, and tumor purity **(D)** between the normal and patients with KIRC in the entire cohort. **(E–H)** Differentially expressed analyses for the ESTIMATE **(E)**, immune **(F)**, stromal **(G)**, and tumor purity **(H)** between the patients with KIRC with low-risk value and high-risk value in the entire cohort. **(I)** Correlation analyses of these five FI-DEGs and risk value with the immune in the entire cohort. N (normal) = 72, N (cancer) = 530. N (low) = 336, N (high) =194. *p < 0.05, ***p < 0.001.

We evaluated the immunity status of the patients with KIRC using these 7,500 DEGs by ESTIMATE in R (3.6.2). The ESTIMATE score, immune score, and stromal score were significantly increased, whereas the tumor purity was significantly decreased in the patients with KIRC (**Figures 5A–D**). Moreover, the ESTIMATE score, immune score, and stromal score were significantly decreased, whereas the tumor purity was significantly increased in the patients with KIRC with high-risk value (**Figures 5E–H**). In addition, the expression of CCR4, MX2, and NR3C2 were significantly correlated with the stromal score, immune score, ESTTIMATE score, and tumor purity (**Figure 5I**). IFITM1 expression was significantly correlated ESTIMATE score and tumor purity (**Figure 5I**).

**Figure 6:**
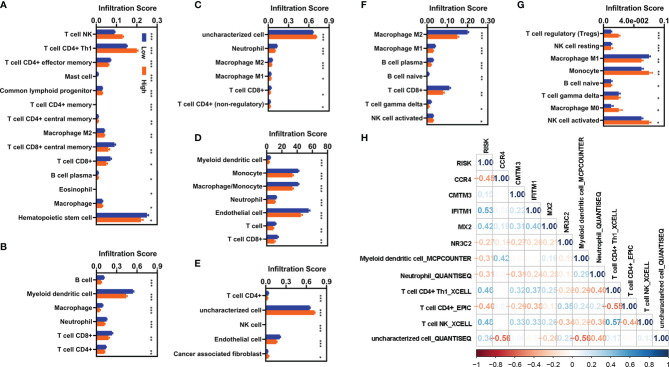
Correlation analyses with the infiltration of immune cells and factors. **(A–G)** Differentially expressed analyses for infiltration of immune cells and factors with different risk value. **(A)** XCELL. **(B)** TIMER. **(C)** QUANTISEQ. **(D)** MCPCOUNTER. **(E)** EPIC. **(F)** CIBERSORT-ABS. **(G)** CIBERSORT. **(H)** Correlation analyses of these five FI-DEGs and risk value with the immune cells and factors in the entire cohort. N (low) = 336, N (high) = 194. *p < 0.05, **p < 0.01, ***p < 0.001.

We also evaluated the relationship of infiltration of immune cells and factors with the risk value. First, we found that there were 88 immune cells and factors whose infiltration values were significantly difference between normal and patients with KIRC (**Supplementary Table 2**). Of which, there were 53 immune cells and factors that are significantly difference between patients with KIRC with low-risk value and high-risk value (**Figures 6A-G**). Correlation analyses showed six immune cells and factors were significantly correlated with the risk value (**Figure 6H**). In addition, these five FI-DEGs were also significantly correlated with several immune and factors of six immune cells and factors (**Figure 6H**).

### Functional Enrichment Analyses

**Figure 7:**
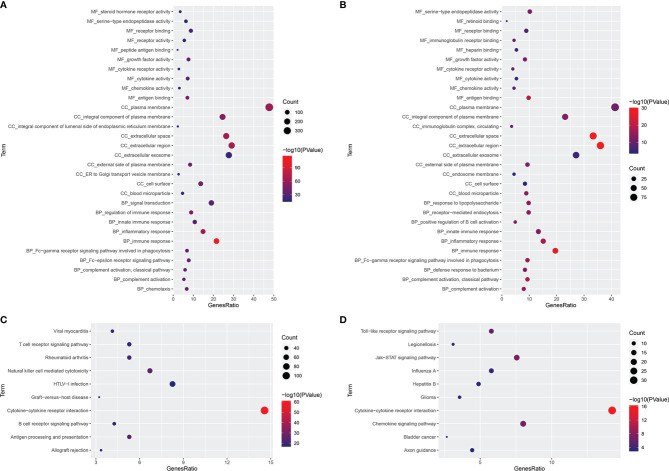
Functional Enrichment Analyses. **(A, B)** The significantly enriched GO term (top 10) for those 784 FI-DGEs **(A)** and those 226 FI-DEGs **(B)**. **(C, D)** The significantly enriched KEGG pathway (top 10) for those 784 FI-DGEs **(C)** and those 226 FI-DEGs **(D)**.

GO and KEGG analyses were carried out for these 784 FI-DEGs between normal and patients with KIRC and 226 FI-DEGs between patients with KIRC with low-risk value and high-risk value (**Supplementary Figure 4**). There were 366 biological processes (BPs), 45 cellular components (CCs), 81 molecular functions (MFs), and 87 KEGG pathways that were enriched as measured by the False discovery rate (FDR) value <0.05 for those 784 FI-DEGs between the normal and patients with KIRC (**Figures 7A, C** and **Supplementary Tables 3, 4**). There were 68 BPs, 11 CCs, 18 MFs, and 16 KEGG pathways that were enriched as measured by the FDR value <0.05 for those 226 FI-DEGs between patients with KIRC with low-risk value and high-risk value (**Figures 7B, D** and **Supplementary Tables 5, 6**).

### Construction of the Diagnostic Model

**Figure 8:**
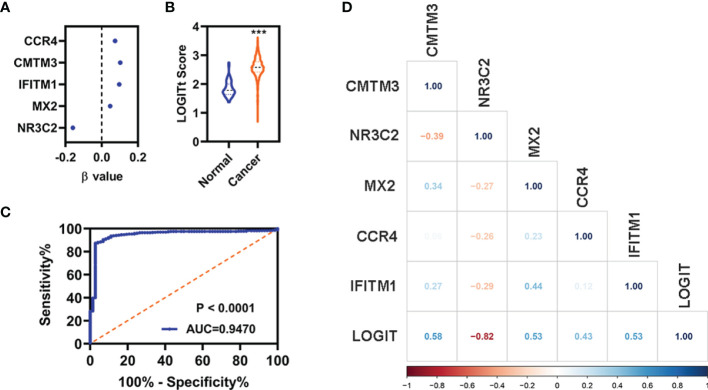
Diagnosis model for distinguishing KIRC from normal in entire cohort. **(A)** The β value of these five FI-DEGs analyses by stepwise logistic regression. **(B)** Diagnosis LOGIT values between normal and patients with KIRC. **(C)** ROC curves for evaluating the predictive performance of the diagnostic model. **(D)** Correlation analyses for the expression of these five FI-DEGs and diagnostic model. N (normal) = 72, N (cancer) = 530. ***p < 0.001.

A diagnostic model integrating these five DEGs (*CCR4*, *CMTM3*, *IFITM1*, *MX2*, and *NR3C2*) were established to separate KIRC from normal using a stepwise logistic regression method. Diagnostic scores were identified as follows: LOGIT score = 0.7998 + (0.1034)*Exp_(CMTM3)_ + (−0.1590)*Exp_(NR3C2)_ + (0.0465)*Exp_(MX2)_ + (0.0737)*Exp_(CCR4)_ + (0.0966)*Exp_(IFITM1)_

(**Figure 8A**). The LOGIT value of patients with KIRC was significantly higher than that of the normal (**Figure 8B**). The AUC value of the diagnostic model reached 0.9470 (**Figure 8C**). Correlation analyses indicated that these five FI-DEGs were significantly correlated the LOGIT value (**Figure 8D**). The sensitivity and specificity of the diagnostic model were 86.98% and 97.22%, respectively (**Table 2**).

**Table 2:** Sensitivity and specificity of diagnosis model.

	Real Cancer	Real Normal
**Predicted Cancer**	461	2
**Predicted Normal**	69	70
**Total**	530	72
**Correct**	461	70
**Sensitivity**	0.8698	
**Specificity**		0.9722

## Discussions

DNA methylation is one of the most common epigenetic modifications, which plays important role in the regulation of the structure and expression of genes. Aberrant DNA methylations may lead to the inactivation of tumor suppressor genes or the activation of oncogenes, which could further lead to the cancerigenesis (9–11). Therefore, researchers have conducted a large number of studies related to the DNA methylation profile of various cancers. The DNA methylated profiles provide insights into the etiology of various cancers for the researcher and clinician in early diagnosis and precise treatment. In the present study, we aimed to identify suitable prognostic biomarkers related with aberrant methylations for KIRC using the TCGA data. We identified that FI-DEGs correlated with aberrant methylations (*CCR4*, *CMTM3*, *IFITM1*, *MX2*, and *NR3C2*) were significantly correlated with the OS of KIRC independently. The prognostic model and diagnosis model constructed by these five FI-DEGs can be well used for the prognosis and diagnosis of KIRC respectively.

CCR4 (C-C Motif Chemokine Receptor 4) is the primary receptor for C-C motif chemokine ligand 17 and C-C motif chemokine ligand 22. Suppression of CCR4 could suppress the migration, invasion, and proliferation for several cancers, such as lung cancer, breast cancer, and leukemia (32–34). Patients with high CCR4 expression have a poorer survival prognosis (35). In the present study, we found that the expression of CCR4 was significantly increased in the patients with KIRC. In addition, previous study also indicated that CCR4 could be a prognostic biomarker and correlated with immune infiltrates in head and neck squamous cell carcinoma (36). CCR4 could be used as a therapeutic target for cancer immunotherapy of several cancers, such as adult T-cell leukemia/lymphoma and cutaneous T-cell lymphomas (37). In our present study, we also found that CCR4 was correlated with the infiltration of several immune cells and factors. However, what is very interesting was that patients with KIRC with high expression of CCR4 displayed better OS. Therefore, we speculate that CCR4 may only be related to the survival of KIRC and does not participate in the development of KIRC. CMTM3 (CKLF-like MARVEL transmembrane domain-containing 3), a member of the CMTM family, was found in several human tumors. In addition to being associated with immunity, CCR4 have also been implicated in iron death-related processes, such as Reactive oxygen species (ROS). Molinaro et al. found that Treg cells in *CCR4*^−/−^ sepsis mice showed reduced inhibition of ROS production by activated neutrophils (38). Hsu et al. found that CCR4 was involved in the regulation of ROS production by IL-20 (39). CMTM3 is closely connected with immune system and associated with sex during tumorgenesis (40). The expression of CMTM3 was decreased in several cancers, such as prostate cancer (41), and hepatic carcinoma (42). Overexpression of CMTM3 could inhibit the proliferation, migration, and invasion for several cancers (41, 42). However, the previous study also demonstrated that CMTM3 was overexpressed in pancreatic cancer (43). Results from the study of Zhou et al. indicated that CMTM3 could promote tumor aggressiveness in pancreatic cancer, and CMTM3 overexpression predicts poor survival (43). CMTM3 could be used as a potential prognostic biomarker of glioma, which is associated with immune invasion in the glioma microenvironment and may become a new immunotherapy target (44). In the present study, we also found that the expression of CMTM3 was increased. Patients with KIRC with high expression of CMTM3 displayed worse OS. All of these results indicated that CMTM3 may serve different role for different cancers. IFITM1 (interferon-induced transmembrane protein 1) is a member of interferon stimulated family, which is expressed by T cells (45). Recent experiments have shown that IFITM protein is directly involved in adaptive immunity and regulates the differentiation of CD4^+^ T helper cells in a T-cell intrinsic manner (45). Results from the study of Lui et al. indicated that IFITM1 overexpression contributes to breast cancer progression (46). Yan et al. found that suppression of IFITM1 could suppress cell growth and metastasis for lung cancer (47). Numerous pieces of evidence indicated that IFITM1 may serve as prognostic biomarker due to the closely relationship with survival (48–50). Consistent with previous studies, we found that the expression of IFITM1 was increased and correlated with the OS of patients with KIRC. MX2 (MX dynamin-like GTPase 2) is a novel regulator of cell cycle in melanoma cells. Wang et al. found the overexpression of human MX2 gene suppresses cell proliferation, migration, and invasion. However, we found that MX2 was reduced in KIRC (51). It may be similar to CMTM3; MX2 may play different roles in different cancers. NR3C2 is nuclear receptor subfamily 3 group C member 2. Fan et al. found that the expression of NR3C2 was downregulated in breast cancer (52). High expression of NR3C2 was significantly correlated with prolonged OS (53). Overexpression NR3C2 could repress the proliferation, migration, and invasion for breast cancer and hepatocellular carcinoma (52, 54). Consistent with previous studies, NR3C2 was decreased in patients with KIRC. Moreover, low NR3C2 correlated with the metastasis and poor prognosis (55). Our studies reinforced the negative relationship of NR3C2 with the KIRC.

## Conclusions

Our present data showed that the prognostic and diagnostic models using those five FI-DEGs (*CCR4*, *CMTM3*, *IFITM1*, *MX2*, and *NR3C2*) could well predict the outcome of patients with KIRC, which suggested that these five FI-DEGs could be used as prognosis and diagnosis biomarkers for KIRC. However, whether these five FI-DEGs are really associated with abnormal methylation and whether these five FI-DEGs can be used for clinical prognosis and diagnosis need further validation, especially clinical cross-validation.

## Data Availability Statement

The data that support the findings of this study are openly available in TCGA at https://portal.gdc.cancer.gov/.

## Author Contributions

ZX and YZ conceived and designed the experiments. X-LX performed the analyses. YL, JL, HFZ, HRZ, QZ, PB, and TD helped to analyze the data. X-LX wrote the paper. All authors contributed to the article and approved the submitted version.

## Funding

This project is financially supported by the Education Department of Hunan Province (20B417) and the Science and Technology Department of Huaihua (2020R3104).

## Conflict of Interest

The authors declare that the research was conducted in the absence of any commercial or financial relationships that could be construed as a potential conflict of interest.

## Publisher’s Note

All claims expressed in this article are solely those of the authors and do not necessarily represent those of their affiliated organizations, or those of the publisher, the editors and the reviewers. Any product that may be evaluated in this article, or claim that may be made by its manufacturer, is not guaranteed or endorsed by the publisher.
