# Supplementary material for: Comprehensive Analysis of Ferroptosis- and Immune-Related Signatures to Improve the Prognosis and Diagnosis of Kidney Renal Clear Cell Carcinoma
Source: Front Immunol. 2022 May 10;13:851312. doi: 10.3389/fimmu.2022.851312 (PMC9128788; doi:10.3389/fimmu.2022.851312)
Supplement: Supplementary file 1 [file DataSheet_1.pdf]

Comprehensive analysis of ferroptosis- and immune-related signatures to improve the prognosis and diagnosis of kidney renal clear cell carcinoma

Xiao-Liang Xing[1,2], Yan Liu[1], Jiheng Liu[1], Huanfa Zhou[1], Huirong Zhang[1], Qi Zuo[3], Ping Bu[1], Tong Duan[3], Yan Zhou[3*], Zhiquan Xiao[1*].

[1]Changsha Central Hospital, University of South China, Changsha, 410004, Hunan P. R. China.

[2]School of Public Health and Laboratory Medicine, Hunan University of Medicine, Huaihua 418000, Hunan P. R. China.

[3]First Hospital of Changsha, Changsha, 410005, Hunan P. R. China.

*Correspondence: Yan Zhou, Email: 921lan@sina.com. Zhiquan Xiao, Email: xzq1892000@163.com.

Supplementary: 4 figures and 6 tables

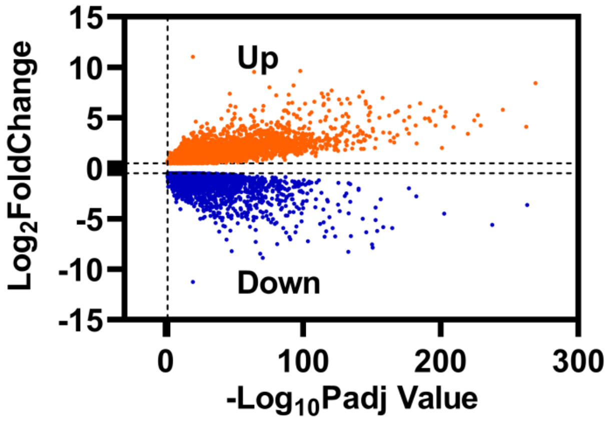

Supplementary Figure 1 Volcano plot of DEGs between normal and patients with KIRC.

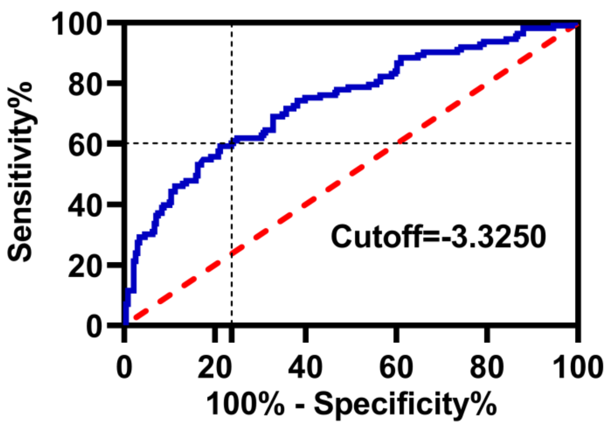

Supplementary Figure 2 Yonden index as the optimal cutoff value.

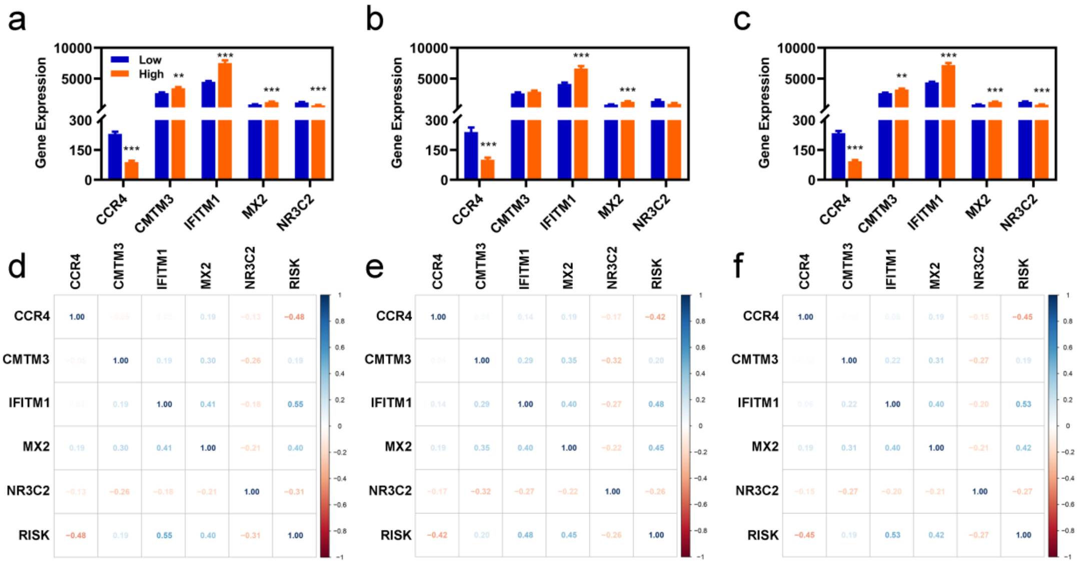

Supplementary Figure 3 Expression of these five FI-DEGs in the different cohort.

a-c, . Expression of these five FI-DEGs in the training cohort (a), validation cohort (b), and entire cohort (c). **d-f,** Correlation of these five FI-DEGs with the risk value in the training cohort (d), validation cohort (e), and entire cohort (f).

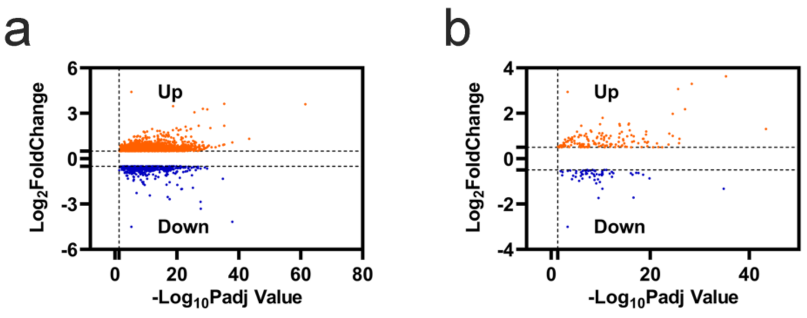

Supplementary Figure 4 Volcano plot of DEGs (a) and FI-DEGs (b) between KIRC patients with low risk value and KIRC patients with high risk value.

Supplementary table 1 Correlated pairs of 256 DMPs and 138 FI-DEGs.

GENE1	GENE2	P	R	GENE1	GENE2	P	R
cg00029282	PROM2	0.00	-0.87	cg15016481	LRRFIP1	0.00	-0.39
cg00146864	MAP3K14	0.00	-0.41	cg15049101	FGFR2	0.00	-0.32
cg00249032	SDC4	0.00	-0.34	cg15066837	GNAI1	0.00	-0.44
cg00331677	CX3CL1	0.00	-0.47	cg15095327	IL17RE	0.00	-0.69
cg00417094	CD247	0.00	-0.33	cg15104031	S100A14	0.00	-0.42
cg00471190	IL32	0.00	-0.32	cg15129144	EPAS1	0.00	-0.55
cg00532455	ACVR2A	0.00	-0.42	cg15281283	MX2	0.00	-0.35
cg00533183	PSMB8	0.00	-0.40	cg15475502	SEMA5B	0.00	-0.41
cg00539360	VEGFA	0.00	-0.63	cg15799353	TRIB3	0.00	-0.55
cg00575003	ESRRG	0.00	-0.39	cg15994182	PROM2	0.00	-0.83
cg00780520	PVT1	0.00	-0.52	cg16001384	NDRG1	0.00	-0.45
cg00798281	HLA-DPA1	0.00	-0.44	cg16172329	DPP4	0.00	-0.32
cg00902427	MCHR1	0.00	-0.35	cg16363586	BST2	0.00	-0.42
cg01059398	TNFSF10	0.00	-0.44	cg16446824	SEMA6A	0.00	-0.55
cg01154445	NRP2	0.00	-0.43	cg16467015	NEDD4L	0.00	-0.53
cg01165683	THRB	0.00	-0.50	cg16486109	IRF7	0.00	-0.45
cg01189606	PLXNB1	0.00	-0.49	cg16592658	EBI3	0.00	-0.32
cg01298514	VEGFA	0.00	-0.59	cg16649560	IL4R	0.00	-0.39
cg01329005	BST2	0.00	-0.33	cg16744911	SCD	0.00	-0.46
cg01356829	IL12RB2	0.00	-0.47	cg16771652	NOD2	0.00	-0.36
cg01673307	TAP1	0.00	-0.54	cg16860392	FYN	0.00	-0.38
cg01843506	CCR4	0.00	-0.40	cg16890093	PSMB8	0.00	-0.47
cg01889574	CD82	0.00	0.41	cg16900589	LRRFIP1	0.00	-0.31
cg01924561	SLC2A1	0.00	-0.43	cg16949914	IL2RA	0.00	-0.30
cg01942646	NR0B2	0.00	-0.36	cg17127769	LCP2	0.00	-0.55
cg02099418	CD28	0.00	-0.40	cg17356733	IFNGR2	0.00	-0.49

cg02117924	ITGB2	0.00	0.31	cg17626301	TAP1	0.00	-0.41
cg02181920	TAP1	0.00	-0.53	cg17864487	LRRFIP1	0.00	-0.50
cg02286081	HLA-DPB1	0.00	-0.48	cg18073906	SEMA5B	0.00	0.44
cg02334775	ISG20	0.00	-0.36	cg18140824	MASP1	0.00	-0.56
cg02365648	BNIP3	0.00	-0.45	cg18367578	NNMT	0.00	-0.35
cg02657012	PSMB8	0.00	-0.48	cg18524262	NEDD4L	0.00	-0.61
cg02774334	SEMA4A	0.00	-0.42	cg18687085	NEDD4L	0.00	0.33
cg02806715	HLA-DMA	0.00	-0.46	cg18696027	B2M	0.00	-0.42
cg03030419	LRRFIP1	0.00	-0.34	cg18739537	NFKBIE	0.00	-0.31
cg03440556	SCD	0.00	-0.46	cg19009305	ISG20	0.00	-0.36
cg03458957	PVT1	0.00	-0.50	cg19076587	PHF21A	0.00	0.35
cg03481855	PVT1	0.00	-0.42	cg19136673	PSMB8	0.00	-0.45
cg03489712	ZYX	0.00	-0.33	cg19170009	VIM	0.00	-0.53
cg03520342	HLA-DMA	0.00	-0.44	cg19248557	HLA-DRA	0.00	-0.37
cg03653601	ABCC1	0.00	-0.51	cg19254514	MASP1	0.00	-0.64
cg03771004	NR1H4	0.00	-0.30	cg19408145	CD48	0.00	-0.32
cg03820120	SEMA6A	0.00	-0.43	cg19516340	RRM2	0.00	-0.30
cg03840259	GRAP2	0.00	-0.37	cg19528338	BMP2	0.00	-0.31
cg03846076	STC2	0.00	-0.48	cg19572487	RARA	0.00	-0.40
cg03885527	PLIN2	0.00	-0.60	cg19728382	STC2	0.00	-0.36
cg03952543	FABP7	0.00	-0.63	cg19789466	OAS1	0.00	-0.37
cg04098585	CD28	0.00	-0.38	cg20136855	TNFRSF1B	0.00	-0.32
cg04171808	CD44	0.00	-0.34	cg20295214	AVPR1B	0.00	-0.45
cg04312209	IL7R	0.00	-0.30	cg20313963	SLC2A3	0.00	-0.31
cg04474049	CAV1	0.00	-0.43	cg20610181	CA9	0.00	-0.58
cg04822621	LCP2	0.00	-0.47	cg20617328	HLA-DPA1	0.00	-0.34
cg04880737	LRRFIP1	0.00	-0.35	cg20703928	NCK2	0.00	-0.42
cg04972436	BIRC5	0.00	0.30	cg20724257	HLA-DRA	0.00	-0.37
cg05253480	IL17RE	0.00	-0.75	cg20808613	AEN	0.00	-0.32
cg05336051	SH3BP2	0.00	-0.44	cg20868817	CD48	0.00	-0.33
cg05372679	SEMA6D	0.00	0.32	cg20940044	VEGFA	0.00	-0.50
cg05412410	CARD11	0.00	0.38	cg21038461	ESRRB	0.00	0.32
cg05437692	NR3C2	0.00	-0.45	cg21126506	ESRRB	0.00	0.36
cg05490029	IL7	0.00	-0.37	cg21139003	LTBP1	0.00	-0.34
cg05656374	MX2	0.00	-0.35	cg21176130	HLA-E	0.00	-0.32
cg05693489	TAP1	0.00	-0.52	cg21211730	CBLC	0.00	-0.32
cg05697726	PSMB8	0.00	-0.46	cg21574271	BMPR1B	0.00	0.43
cg05989861	PROM2	0.00	-0.82	cg21681212	EGFR	0.00	-0.33
cg06159340	CTF1	0.00	-0.65	cg21686213	IFITM1	0.00	-0.44
cg06473288	TAP1	0.00	-0.54	cg21708130	LRRFIP1	0.00	0.32
cg06583577	SEMA6A	0.00	-0.36	cg21758773	HLA-E	0.00	-0.37
cg06650260	NR0B2	0.00	-0.50	cg21784383	ESRRG	0.00	-0.31
cg06656414	SEMA5B	0.00	-0.49	cg21831174	MASP1	0.00	-0.58

cg07044282	ANGPTL1	0.00	-0.57	cg21896394	PVT1	0.00	-0.39
cg07151443	HLA-DMA	0.00	-0.33	cg22193276	LPIN1	0.00	-0.45
cg07355189	ALOX5	0.00	0.36	cg22198044	SH3BP2	0.00	0.41
cg07784959	CXCR4	0.00	-0.43	cg22272100	BMP1	0.00	-0.30
cg07991621	SH3BP2	0.00	0.36	cg22298860	HLA-F	0.00	-0.31
cg08110861	APOM	0.00	-0.35	cg22309950	CD28	0.00	-0.57
cg08173915	IFNGR2	0.00	-0.50	cg22427313	EGFR	0.00	-0.33
cg08246632	ULK1	0.00	-0.30	cg22572614	TNFSF10	0.00	-0.32
cg08575688	CCL20	0.00	-0.33	cg22920258	MCHR1	0.00	-0.34
cg09077096	CARD11	0.00	0.45	cg23071186	TNFSF14	0.00	-0.31
cg09234582	HLA-DPB1	0.00	0.35	cg23084016	ALOX5	0.00	-0.36
cg09321817	HLA-DPA1	0.00	-0.44	cg23114435	CD247	0.00	-0.44
cg09326440	HLA-E	0.00	-0.36	cg23251761	PIK3CD	0.00	0.57
cg09354037	TAP1	0.00	-0.56	cg23256150	NR1H4	0.00	-0.30
cg09664492	PROM2	0.00	-0.80	cg23295629	PVT1	0.00	-0.31
cg09807033	SEMA6A	0.00	-0.45	cg23297477	CMTM3	0.00	-0.30
cg09826050	ESRRG	0.00	-0.42	cg23374992	CXCR4	0.00	-0.49
cg09837648	PLXNB1	0.00	-0.46	cg23570810	IFITM1	0.00	-0.32
cg09993699	BST2	0.00	-0.32	cg23613051	SH3BP2	0.00	0.40
cg10009968	CARD11	0.00	0.38	cg23656322	S100A2	0.00	0.32
cg10055471	NR0B2	0.00	-0.57	cg23821329	VIM	0.00	-0.45
cg10093934	PDK1	0.00	-0.32	cg23923934	HLA-B	0.00	-0.58
cg10224600	MAPT	0.00	0.41	cg24111025	TAP1	0.00	-0.55
cg10230442	HRG	0.00	-0.32	cg24129356	HLA-DMA	0.00	-0.50
cg10316474	APOBEC3C	0.00	-0.53	cg24302529	ALOX5	0.00	0.37
cg10362335	TNFSF14	0.00	-0.38	cg24421410	HLA-DMA	0.00	-0.46
cg10522125	ITGAL	0.00	-0.47	cg24443250	TNFSF8	0.00	-0.35
cg10601943	HLA-F	0.00	-0.52	cg24476033	C3	0.00	0.31
cg10745272	SH3BP2	0.00	0.38	cg24492202	SH3BP2	0.00	-0.53
cg10790685	VIM	0.00	-0.44	cg24512973	MUC1	0.00	-0.61
cg10864979	PVT1	0.00	-0.34	cg24580782	NR0B2	0.00	-0.62
cg10869531	SH3BP2	0.00	-0.52	cg24593324	VCAM1	0.00	-0.46
cg11145461	CSRP1	0.00	-0.40	cg25018049	CSRP1	0.00	-0.37
cg11215296	AVPR1B	0.00	-0.48	cg25052156	FGFR2	0.00	-0.52
cg11909912	MAPT	0.00	0.38	cg25135018	IL6R	0.00	-0.39
cg12069042	PLXNB1	0.00	-0.53	cg25198340	IL17RD	0.00	-0.42
cg12123019	IL12RB1	0.00	-0.49	cg25241038	PROM2	0.00	-0.82
cg12253437	TAP1	0.00	-0.54	cg25258233	HLA-DRA	0.00	-0.35
cg12595667	CXCR4	0.00	-0.41	cg25281029	KITLG	0.00	-0.44
cg12700271	HLA-E	0.00	-0.33	cg25343661	VEGFA	0.00	-0.59
cg12710152	LCK	0.00	-0.39	cg25373579	VEGFA	0.00	-0.61
cg12939283	HLA-DPB1	0.00	-0.40	cg25580782	IL15RA	0.00	0.30
cg13007871	HLA-E	0.00	-0.37	cg25592413	STC2	0.00	-0.44

cg13092901	TYMP	0.00	-0.40	cg25594165	NRG3	0.00	-0.35
cg13144783	CCR1	0.00	-0.32	cg25647415	SEMA3B	0.00	-0.30
cg13165140	TAP1	0.00	-0.57	cg25954539	HLA-B	0.00	-0.46
cg13346441	BID	0.00	0.32	cg25961567	NCK2	0.00	-0.48
cg13445177	S100A10	0.00	-0.30	cg26063719	VIM	0.00	-0.56
cg13493001	LCN12	0.00	-0.41	cg26065841	CHAC1	0.00	-0.38
cg13592599	JAG1	0.00	-0.40	cg26234900	TAP1	0.00	-0.36
cg13727618	INSR	0.00	-0.50	cg26315984	TNFSF4	0.00	-0.34
cg14052728	PROM2	0.00	-0.81	cg26351966	SCD	0.00	-0.37
cg14082886	CD44	0.00	-0.34	cg26389955	PRKCA	0.00	0.36
cg14260889	VIM	0.00	-0.56	cg26549174	ICAM1	0.00	-0.36
cg14328173	MASP1	0.00	-0.55	cg26675785	ABCC1	0.00	-0.30
cg14334810	PLIN2	0.00	-0.51	cg26815454	GRAP2	0.00	-0.31
cg14356799	HLA-DPB1	0.00	-0.43	cg26878655	RARA	0.00	-0.43
cg14520913	NNMT	0.00	-0.38	cg26928531	HLA-DRA	0.00	-0.31
cg14967066	IFITM1	0.00	-0.41	cg27544046	VCAM1	0.00	-0.34

Supplementary table 2 The difference immune cells and factors between normal and KIRC patients.

**Type**	**Cell**	**Normal (n=72)**		**Cancer (n=530)**	
		**MEAN**	**STD**	**MEAN**	**STD**
CIBERSORT	B cell naive	0.09	0.05	0.01	0.02
	T cell CD4+ memory resting	0.23	0.06	0.13	0.08
	Macrophage M1	0.03	0.02	0.06	0.03
	T cell CD8+	0.05	0.04	0.17	0.12
	T cell follicular helper	0.01	0.01	0.03	0.03
	Mast cell activated	0.05	0.05	0.02	0.03
	B cell plasma	0.08	0.06	0.05	0.04
	Monocyte	0.08	0.05	0.05	0.05
	Macrophage M2	0.26	0.10	0.33	0.12
	T cell gamma delta	0.00	0.01	0.02	0.03
	T cell regulatory (Tregs)	0.01	0.01	0.01	0.02
	NK cell resting	0.00	0.01	0.01	0.02
	Macrophage M0	0.00	0.01	0.02	0.06
	Eosinophil	0.00	0.00	0.00	0.00
	NK cell activated	0.06	0.04	0.05	0.03
	Mast cell resting	0.02	0.04	0.01	0.03
CIBERSORT-ABS	B cell naive	0.02	0.01	0.01	0.01
	Macrophage M2	0.07	0.06	0.18	0.10
	Macrophage M1	0.01	0.01	0.03	0.03
	T cell CD8+	0.01	0.01	0.10	0.09

	T cell follicular helper	0.00	0.00	0.02	0.02
	NK cell activated	0.01	0.01	0.03	0.02
	T cell regulatory (Tregs)	0.00	0.00	0.01	0.01
	T cell gamma delta	0.00	0.00	0.01	0.02
	B cell plasma	0.02	0.01	0.03	0.02
	Macrophage M0	0.00	0.00	0.01	0.02
	NK cell resting	0.00	0.00	0.00	0.01
EPIC	T cell CD4+	0.09	0.02	0.04	0.02
	Endothelial cell	0.07	0.05	0.19	0.12
	Macrophage	0.00	0.00	0.03	0.03
	uncharacterized cell	0.79	0.05	0.68	0.14
	NK cell	0.00	0.00	0.00	0.00
	Cancer associated fibroblast	0.01	0.01	0.03	0.05
MCPCOUNTER	T cell	63.42	34.48	10.51	11.49
	Monocyte	14.54	8.67	38.57	17.33
	Macrophage/Monocyte	14.54	8.67	38.57	17.33
	NK cell	0.18	0.17	0.66	0.37
	Neutrophil	18.87	6.46	12.07	5.30
	cytotoxicity score	1.12	1.33	9.06	7.24
	Endothelial cell	24.34	10.13	51.37	30.49
	T cell CD8+	1.36	1.42	12.84	17.05
	Myeloid dendritic cell	2.78	2.05	4.24	2.51
QUANTISEQ	Macrophage M1	0.01	0.01	0.04	0.03
	T cell CD4+ (non-regulatory)	0.01	0.01	0.03	0.02
	Neutrophil	0.17	0.08	0.12	0.05
	T cell CD8+	0.00	0.00	0.03	0.05
	Macrophage M2	0.04	0.01	0.06	0.03
	uncharacterized cell	0.73	0.06	0.68	0.08
	T cell regulatory (Tregs)	0.01	0.01	0.01	0.01
	Myeloid dendritic cell	0.02	0.02	0.01	0.02
	NK cell	0.01	0.00	0.01	0.01
TIMER	Neutrophil	0.03	0.04	0.14	0.10
	Myeloid dendritic cell	0.28	0.12	0.50	0.22
	T cell CD8+	0.04	0.05	0.21	0.18
	T cell CD4+	0.10	0.05	0.14	0.08
	Macrophage	0.03	0.06	0.08	0.11
	B cell	0.07	0.05	0.10	0.07
XCELL	T cell NK	0.01	0.01	0.11	0.05
	T cell CD4+ effector memory	0.00	0.01	0.07	0.04
	Myeloid dendritic cell activated	0.07	0.08	0.21	0.09
	Hematopoietic stem cell	0.47	0.15	0.24	0.15
	microenvironment score	0.10	0.05	0.25	0.11
	T cell CD4+ Th1	0.09	0.03	0.17	0.07

	Endothelial cell	0.10	0.05	0.23	0.12
	Monocyte	0.00	0.01	0.04	0.04
	immune score	0.01	0.01	0.12	0.11
	Mast cell	0.00	0.00	0.01	0.01
	T cell CD8+ naive	0.00	0.00	0.01	0.01
	T cell CD8+ central memory	0.00	0.01	0.08	0.09
	Macrophage	0.00	0.01	0.03	0.04
	T cell CD8+	0.00	0.00	0.07	0.09
	T cell CD8+ effector memory	0.00	0.00	0.03	0.05
	Macrophage M1	0.00	0.00	0.01	0.02
	Macrophage M2	0.02	0.01	0.04	0.03
	Myeloid dendritic cell	0.00	0.01	0.01	0.01
	stroma score	0.09	0.05	0.13	0.07
	B cell plasma	0.00	0.00	0.01	0.02
	Class-switched memory B cell	0.00	0.00	0.01	0.01
	B cell	0.00	0.00	0.02	0.05
	Plasmacytoid dendritic cell	0.00	0.00	0.01	0.01
	T cell CD4+ central memory	0.00	0.00	0.01	0.01
	T cell CD4+ Th2	0.00	0.02	0.03	0.07
	Cancer associated fibroblast	0.04	0.07	0.02	0.05
	T cell CD4+ naive	0.00	0.00	0.01	0.02
	B cell naive	0.00	0.00	0.00	0.00
	T cell CD4+ memory	0.00	0.00	0.00	0.01
	Common lymphoid progenitor	0.03	0.01	0.03	0.02
	Eosinophil	0.00	0.00	0.00	0.00

Supplementary table 3 The significantly enriched GO term for 784 FI-DEGs between the normal and patients with KIRC.

Category_Term		Count	FDR
BP	immune response	167	0.0000
	inflammatory response	115	0.0000
	regulation of immune response	69	0.0000
	Fc-gamma receptor signaling pathway involved in phagocytosis	53	0.0000
	chemotaxis	52	0.0000
	Fc-epsilon receptor signaling pathway	60	0.0000
	complement activation, classical pathway	46	0.0000
	signal transduction	147	0.0000
	complement activation	41	0.0000
	innate immune response	83	0.0000
	response to lipopolysaccharide	51	0.0000
	positive regulation of ERK1 and ERK2 cascade	52	0.0000

	cell surface receptor signaling pathway	64	0.0000
	positive regulation of cell proliferation	83	0.0000
	receptor-mediated endocytosis	53	0.0000
	cell-cell signaling	61	0.0000
	interferon-gamma-mediated signaling pathway	34	0.0000
	chemokine-mediated signaling pathway	33	0.0000
	T cell costimulation	34	0.0000
	angiogenesis	52	0.0000
	positive regulation of cell migration	46	0.0000
	positive regulation of angiogenesis	37	0.0000
	semaphorin-plexin signaling pathway	22	0.0000
	positive regulation of cytosolic calcium ion concentration	39	0.0000
	steroid hormone mediated signaling pathway	27	0.0000
	response to hypoxia	43	0.0000
	B cell receptor signaling pathway	26	0.0000
	cell chemotaxis	28	0.0000
	T cell receptor signaling pathway	39	0.0000
	antigen processing and presentation	25	0.0000
	neutrophil chemotaxis	26	0.0000
	transmembrane receptor protein tyrosine kinase signaling pathway	30	0.0000
	positive regulation of peptidyl-tyrosine phosphorylation	28	0.0000
	positive regulation of phosphatidylinositol 3-kinase signaling	25	0.0000
	regulation of cell proliferation	40	0.0000
	positive regulation of MAPK cascade	27	0.0000
	negative regulation of apoptotic process	64	0.0000
	negative regulation of axon extension involved in axon guidance	17	0.0000
	positive regulation of endothelial cell proliferation	25	0.0000
	leukocyte migration	32	0.0000
	tumor necrosis factor-mediated signaling pathway	31	0.0000
	MAPK cascade	46	0.0000
	negative chemotaxis	18	0.0000
	positive regulation of B cell activation	16	0.0000
	regulation of apoptotic process	40	0.0000
	defense response to virus	35	0.0000
	positive regulation of NF-kappaB transcription factor activity	31	0.0000
	negative regulation of cell proliferation	55	0.0000
	peptidyl-tyrosine phosphorylation	33	0.0000
	type I interferon signaling pathway	22	0.0000
	positive regulation of transcription from RNA polymerase II promoter	96	0.0000
	antigen processing and presentation of peptide antigen via MHC class I	16	0.0000
	positive regulation of inflammatory response	23	0.0000

	positive regulation of cell division	19	0.0000
	positive regulation of pathway-restricted SMAD protein phosphorylation	19	0.0000
	positive regulation of protein kinase B signaling	24	0.0000
	positive regulation of protein phosphorylation	29	0.0000
	positive regulation of gene expression	42	0.0000
	semaphorin-plexin signaling pathway involved in axon guidance	11	0.0000
	phosphatidylinositol-mediated signaling	26	0.0000
	positive chemotaxis	16	0.0000
	defense response to bacterium	30	0.0000
	antigen processing and presentation of peptide or polysaccharide antigen via MHC class II	12	0.0000
	cytokine-mediated signaling pathway	28	0.0000
	cellular response to lipopolysaccharide	26	0.0000
	intracellular receptor signaling pathway	16	0.0000
	neural crest cell migration	17	0.0000
	positive regulation of MAP kinase activity	19	0.0000
	positive regulation of smooth muscle cell proliferation	19	0.0000
	positive regulation of T cell proliferation	19	0.0000
	response to virus	25	0.0000
	T cell activation	17	0.0000
	positive regulation of vasodilation	14	0.0000
	phosphatidylinositol phosphorylation	23	0.0000
	regulation of phosphatidylinositol 3-kinase signaling	21	0.0000
	branchiomotor neuron axon guidance	9	0.0000
	monocyte chemotaxis	16	0.0000
	cellular response to mechanical stimulus	20	0.0000
	apoptotic signaling pathway	20	0.0000
	vascular endothelial growth factor receptor signaling pathway	20	0.0000
	cellular response to interferon-gamma	18	0.0000
	adaptive immune response	28	0.0000
	phagocytosis, recognition	13	0.0000
	proteolysis	55	0.0000
	wound healing	20	0.0000
	phagocytosis, engulfment	14	0.0000
	cellular response to tumor necrosis factor	23	0.0000
	defense response	18	0.0000
	lipopolysaccharide-mediated signaling pathway	13	0.0000
	platelet activation	23	0.0000
	apoptotic process	58	0.0000
	positive regulation of interferon-gamma production	15	0.0000
	SMAD protein signal transduction	17	0.0000
	antigen processing and presentation of exogenous peptide antigen	8	0.0000

	via MHC class I, TAP-independent		
	transcription initiation from RNA polymerase II promoter	26	0.0000
	macrophage chemotaxis	9	0.0000
	G-protein coupled receptor signaling pathway	78	0.0000
	positive regulation of apoptotic process	38	0.0000
	activation of MAPK activity	21	0.0000
	positive regulation of phosphatidylinositol 3-kinase activity	12	0.0000
	regulation of axon extension involved in axon guidance	7	0.0000
	positive regulation of endothelial cell migration	14	0.0000
	positive regulation of fibroblast proliferation	15	0.0000
	aging	26	0.0000
	induction of positive chemotaxis	9	0.0000
	response to wounding	16	0.0000
	peptidyl-tyrosine autophosphorylation	13	0.0000
	epidermal growth factor receptor signaling pathway	15	0.0000
	cell proliferation	41	0.0000
	extrinsic apoptotic signaling pathway	13	0.0000
	growth	11	0.0000
	T cell chemotaxis	7	0.0000
	receptor internalization	13	0.0000
	positive regulation of epithelial cell proliferation	15	0.0000
	cellular response to vascular endothelial growth factor stimulus	10	0.0000
	T cell differentiation	11	0.0000
	cellular response to interleukin-1	16	0.0000
	positive regulation of leukocyte chemotaxis	9	0.0000
	ureteric bud development	12	0.0000
	antigen processing and presentation of exogenous peptide antigen via MHC class II	18	0.0000
	defense response to Gram-negative bacterium	14	0.0000
	activation of cysteine-type endopeptidase activity involved in apoptotic process	17	0.0000
	response to drug	35	0.0000
	cytokine production	10	0.0000
	positive regulation of blood vessel endothelial cell migration	9	0.0000
	defense response to Gram-positive bacterium	17	0.0000
	negative regulation of viral genome replication	12	0.0000
	retina homeostasis	12	0.0000
	positive regulation of mitotic nuclear division	10	0.0000
	positive regulation of extrinsic apoptotic signaling pathway	10	0.0000
	positive regulation of GTPase activity	52	0.0000
	positive regulation of DNA replication	12	0.0000
	negative regulation of endothelial cell apoptotic process	10	0.0001
	lymphocyte chemotaxis	10	0.0001

	response to estradiol	17	0.0001
	cellular iron ion homeostasis	12	0.0001
	antibacterial humoral response	12	0.0001
	positive regulation of I-kappaB kinase/NF-kappaB signaling	23	0.0001
	negative regulation of gene expression	21	0.0001
	negative regulation of angiogenesis	14	0.0001
	positive regulation of neutrophil chemotaxis	9	0.0001
	immunoglobulin mediated immune response	7	0.0001
	antigen processing and presentation of exogenous peptide antigen via MHC class I, TAP-dependent	14	0.0001
	positive regulation of interleukin-6 production	12	0.0001
	antigen processing and presentation of endogenous peptide antigen via MHC class I	6	0.0001
	stimulatory C-type lectin receptor signaling pathway	18	0.0001
	regulation of cell migration	15	0.0001
	negative regulation of blood pressure	10	0.0001
	positive regulation of tyrosine phosphorylation of Stat3 protein	11	0.0001
	response to glucocorticoid	14	0.0001
	cellular response to hypoxia	17	0.0001
	positive regulation of tumor necrosis factor production	12	0.0001
	response to amino acid	10	0.0001
	response to progesterone	11	0.0001
	positive regulation of B cell proliferation	11	0.0001
	response to heat	12	0.0001
	negative regulation of cell growth	19	0.0001
	positive regulation of vasoconstriction	10	0.0002
	axon guidance	22	0.0002
	leukocyte cell-cell adhesion	9	0.0002
	positive regulation of interleukin-12 production	9	0.0002
	positive regulation of T-helper 2 cell differentiation	6	0.0002
	positive regulation of protein kinase C signaling	6	0.0002
	regulation of MAPK cascade	11	0.0002
	negative regulation of extrinsic apoptotic signaling pathway via death domain receptors	10	0.0002
	intracellular signal transduction	39	0.0002
	viral process	32	0.0002
	detection of bacterium	7	0.0002
	positive regulation of transcription, DNA-templated	46	0.0002
	platelet degranulation	17	0.0002
	protein phosphorylation	42	0.0003
	positive regulation of nitric oxide biosynthetic process	11	0.0003
	positive regulation of vascular endothelial growth factor production	9	0.0003

	positive regulation of activated T cell proliferation	9	0.0003
	positive regulation of interleukin-4 production	8	0.0003
	facial nerve structural organization	6	0.0004
	positive regulation of alpha-beta T cell proliferation	6	0.0004
	trigeminal nerve structural organization	5	0.0004
	response to toxic substance	15	0.0004
	regulation of defense response to virus by virus	9	0.0004
	negative regulation of neuron apoptotic process	19	0.0004
	protein autophosphorylation	22	0.0005
	cell migration involved in sprouting angiogenesis	7	0.0005
	phospholipase C-activating G-protein coupled receptor signaling pathway	13	0.0005
	organ regeneration	11	0.0006
	extrinsic apoptotic signaling pathway via death domain receptors	10	0.0006
	female pregnancy	15	0.0006
	negative regulation of inflammatory response	14	0.0007
	activin receptor signaling pathway	7	0.0007
	positive regulation of vascular endothelial growth factor receptor signaling pathway	7	0.0007
	positive regulation of macroautophagy	8	0.0008
	regulation of platelet aggregation	5	0.0010
	cellular response to iron ion	5	0.0010
	adenylate cyclase-activating G-protein coupled receptor signaling pathway	11	0.0010
	positive regulation of chemokine production	7	0.0011
	mammary gland alveolus development	7	0.0011
	positive regulation of macrophage chemotaxis	6	0.0011
	negative thymic T cell selection	6	0.0011
	cellular defense response	12	0.0013
	positive regulation of phosphorylation	8	0.0014
	response to cytokine	11	0.0014
	response to vitamin D	7	0.0015
	response to nutrient	13	0.0016
	positive regulation of interleukin-8 production	8	0.0017
	negative regulation of bone resorption	6	0.0017
	cellular response to nutrient levels	6	0.0017
	T-helper 1 type immune response	6	0.0017
	PERK-mediated unfolded protein response	6	0.0017
	axon extension involved in axon guidance	6	0.0017
	positive regulation of interleukin-2 biosynthetic process	6	0.0017
	skeletal system development	18	0.0019
	positive regulation of bone mineralization	9	0.0019
	BMP signaling pathway	13	0.0019

	regulation of blood pressure	12	0.0019
	positive regulation of dendritic cell antigen processing and presentation	5	0.0020
	cellular response to hormone stimulus	10	0.0021
	positive regulation of calcium-mediated signaling	7	0.0027
	positive regulation of smooth muscle cell migration	7	0.0027
	patterning of blood vessels	8	0.0027
	negative regulation of T cell proliferation	9	0.0028
	heart development	21	0.0028
	humoral immune response	11	0.0028
	positive regulation of focal adhesion assembly	7	0.0035
	positive regulation of NF-kappaB import into nucleus	7	0.0035
	cellular response to organic cyclic compound	11	0.0037
	superoxide anion generation	6	0.0037
	prostaglandin biosynthetic process	6	0.0037
	phosphatidylinositol-3-phosphate biosynthetic process	10	0.0040
	cellular response to transforming growth factor beta stimulus	10	0.0040
	iron ion homeostasis	8	0.0041
	I-kappaB kinase/NF-kappaB signaling	11	0.0041
	cell development	9	0.0047
	semaphorin-plexin signaling pathway involved in neuron projection guidance	4	0.0050
	neutrophil activation involved in immune response	4	0.0050
	ventral trunk neural crest cell migration	4	0.0050
	sympathetic neuron projection guidance	4	0.0050
	sympathetic neuron projection extension	4	0.0050
	protein refolding	6	0.0051
	positive regulation of cellular protein metabolic process	6	0.0051
	calcium-mediated signaling	10	0.0052
	response to retinoic acid	9	0.0054
	positive regulation of interleukin-10 production	7	0.0056
	response to bacterium	7	0.0056
	positive regulation of chemokine secretion	5	0.0058
	response to molecule of bacterial origin	5	0.0058
	positive regulation of monocyte chemotaxis	6	0.0069
	astrocyte development	6	0.0069
	neurotrophin TRK receptor signaling pathway	6	0.0069
	response to interferon-gamma	7	0.0071
	positive regulation of cell adhesion	9	0.0073
	cell redox homeostasis	12	0.0076
	lipid metabolic process	18	0.0082
	epithelial to mesenchymal transition	8	0.0085
	sprouting angiogenesis	7	0.0087

	positive regulation of cytokine secretion	7	0.0087
	positive regulation of T cell migration	5	0.0089
	negative regulation of hormone secretion	5	0.0089
	dendritic cell chemotaxis	6	0.0090
	lymph node development	6	0.0090
	positive regulation of myoblast fusion	6	0.0090
	blood circulation	9	0.0096
	triglyceride homeostasis	7	0.0106
	peptide antigen assembly with MHC class II protein complex	4	0.0107
	positive regulation of transcription from RNA polymerase II promoter in response to oxidative stress	4	0.0107
	dichotomous subdivision of terminal units involved in salivary gland branching	4	0.0107
	neural crest cell migration involved in autonomic nervous system development	4	0.0107
	negative regulation of cysteine-type endopeptidase activity involved in apoptotic process	11	0.0113
	positive regulation of T cell activation	6	0.0116
	toll-like receptor 4 signaling pathway	6	0.0116
	positive regulation of peptidyl-serine phosphorylation	11	0.0126
	positive regulation of release of sequestered calcium ion into cytosol	7	0.0126
	positive regulation of positive chemotaxis	5	0.0126
	leukocyte migration involved in inflammatory response	5	0.0126
	positive regulation of mast cell degranulation	5	0.0126
	negative regulation of cell adhesion	8	0.0133
	defense response to protozoan	6	0.0148
	T cell proliferation	6	0.0148
	positive regulation of release of cytochrome c from mitochondria	7	0.0151
	regulation of transcription from RNA polymerase II promoter in response to hypoxia	7	0.0151
	TRIF-dependent toll-like receptor signaling pathway	7	0.0151
	positive regulation of osteoblast differentiation	10	0.0151
	ERBB2 signaling pathway	8	0.0153
	movement of cell or subcellular component	12	0.0173
	negative regulation of immune response	5	0.0174
	I-kappaB phosphorylation	5	0.0174
	activation of transmembrane receptor protein tyrosine kinase activity	5	0.0174
	negative regulation of blood coagulation	5	0.0174
	positive regulation of chemotaxis	5	0.0174
	acute-phase response	8	0.0174
	negative regulation of smooth muscle cell proliferation	7	0.0174

	B cell activation	7	0.0174
	homeostasis of number of cells within a tissue	7	0.0174
	negative regulation of endothelial cell proliferation	7	0.0174
	endothelial cell migration	7	0.0174
	liver regeneration	7	0.0174
	decidualization	6	0.0177
	positive regulation of pri-miRNA transcription from RNA polymerase II promoter	6	0.0177
	cellular response to prostaglandin D stimulus	4	0.0181
	regulation of adaptive immune response	4	0.0181
	leukotriene signaling pathway	4	0.0181
	positive regulation of cAMP metabolic process	4	0.0181
	positive regulation of mast cell chemotaxis	4	0.0181
	positive regulation of receptor binding	4	0.0181
	negative regulation of viral process	4	0.0181
	vascular endothelial growth factor signaling pathway	4	0.0181
	positive regulation of type 2 immune response	4	0.0181
	cell migration	18	0.0186
	glucose homeostasis	13	0.0188
	negative regulation of transcription from RNA polymerase II promoter	50	0.0198
	cellular response to fibroblast growth factor stimulus	7	0.0198
	positive regulation of reactive oxygen species metabolic process	7	0.0198
	response to testosterone	7	0.0198
	calcium ion transport	11	0.0206
	positive regulation of axonogenesis	6	0.0211
	cytolysis	6	0.0211
	positive regulation of nitric-oxide synthase biosynthetic process	5	0.0213
	acute inflammatory response	5	0.0213
	positive regulation of interleukin-17 production	5	0.0213
	regulation of peptidyl-tyrosine phosphorylation	5	0.0213
	lipoxygenase pathway	5	0.0213
	pathway-restricted SMAD protein phosphorylation	5	0.0213
	response to yeast	5	0.0213
	positive regulation of T cell mediated cytotoxicity	5	0.0213
	regulation of myelination	5	0.0213
	activation of cysteine-type endopeptidase activity involved in apoptotic signaling pathway	5	0.0213
	positive regulation of urine volume	5	0.0213
	positive regulation of macrophage differentiation	5	0.0213
	positive regulation of JNK cascade	10	0.0230
	embryo implantation	8	0.0241
	cellular response to cytokine stimulus	6	0.0251

	transforming growth factor beta receptor signaling pathway	12	0.0256
	organ morphogenesis	12	0.0256
	regulation of cell adhesion	8	0.0273
	response to insulin	10	0.0276
	humoral immune response mediated by circulating immunoglobulin	4	0.0276
	positive regulation of axon extension involved in axon guidance	4	0.0276
	retina vasculature development in camera-type eye	4	0.0276
	positive regulation of interleukin-13 production	4	0.0276
	response to dexamethasone	4	0.0276
	positive regulation of endothelial cell apoptotic process	5	0.0277
	branching morphogenesis of an epithelial tube	6	0.0299
	positive regulation of collagen biosynthetic process	6	0.0299
	protein kinase B signaling	7	0.0300
	cholesterol metabolic process	10	0.0300
	negative regulation of neuron projection development	8	0.0301
	signal transduction by protein phosphorylation	8	0.0301
	cell growth	9	0.0310
	inflammatory response to antigenic stimulus	5	0.0356
	positive regulation of erythrocyte differentiation	6	0.0356
	positive regulation of epidermal growth factor receptor signaling pathway	6	0.0356
	response to calcium ion	9	0.0383
	activation of MAPKK activity	8	0.0383
	reactive oxygen species metabolic process	7	0.0397
	negative regulation of collagen biosynthetic process	4	0.0405
	regulation of T cell differentiation	4	0.0405
	mast cell activation	4	0.0405
	negative regulation of transposition	4	0.0405
	regulation of type B pancreatic cell proliferation	4	0.0405
	response to tumor necrosis factor	6	0.0418
	intrinsic apoptotic signaling pathway in response to DNA damage	8	0.0422
	positive regulation of protein kinase activity	8	0.0422
	positive regulation of calcium ion import	5	0.0439
	response to muscle stretch	5	0.0439
	positive regulation of macrophage derived foam cell differentiation	5	0.0439
	activation of phospholipase C activity	6	0.0489
	activation of protein kinase B activity	6	0.0489
CC	extracellular region	225	0.0000
	extracellular space	204	0.0000
	plasma membrane	370	0.0000
	integral component of plasma membrane	190	0.0000

	cell surface	106	0.0000
	external side of plasma membrane	65	0.0000
	extracellular exosome	214	0.0000
	blood microparticle	36	0.0000
	integral component of lumenal side of endoplasmic reticulum membrane	18	0.0000
	ER to Golgi transport vesicle membrane	21	0.0000
	MHC class II protein complex	15	0.0000
	membrane raft	38	0.0000
	receptor complex	28	0.0000
	MHC class I protein complex	10	0.0000
	semaphorin receptor complex	10	0.0000
	membrane	147	0.0000
	T cell receptor complex	10	0.0000
	immunoglobulin complex, circulating	10	0.0000
	endocytic vesicle membrane	16	0.0000
	integral component of membrane	276	0.0000
	platelet alpha granule lumen	14	0.0000
	perinuclear region of cytoplasm	53	0.0000
	phagocytic vesicle membrane	13	0.0001
	clathrin-coated endocytic vesicle membrane	11	0.0001
	cytosol	182	0.0002
	focal adhesion	36	0.0002
	alpha-beta T cell receptor complex	5	0.0002
	endosome membrane	21	0.0010
	extrinsic component of cytoplasmic side of plasma membrane	12	0.0012
	transport vesicle membrane	9	0.0016
	HFE-transferrin receptor complex	5	0.0022
	immunological synapse	8	0.0046
	basolateral plasma membrane	19	0.0050
	Golgi membrane	42	0.0076
	lysosomal membrane	24	0.0099
	endosome	21	0.0102
	protein complex	31	0.0172
	Golgi lumen	12	0.0188
	apical plasma membrane	24	0.0196
	phosphatidylinositol 3-kinase complex	5	0.0196
	lysosomal lumen	11	0.0228
	early endosome membrane	13	0.0258
	caveola	9	0.0428
	B cell receptor complex	3	0.0428
	TAP complex	3	0.0428
MF	antigen binding	55	0.0000

	growth factor activity	59	0.0000
	cytokine activity	56	0.0000
	receptor binding	68	0.0000
	steroid hormone receptor activity	27	0.0000
	cytokine receptor activity	22	0.0000
	chemokine activity	24	0.0000
	serine-type endopeptidase activity	49	0.0000
	receptor activity	43	0.0000
	peptide antigen binding	17	0.0000
	phosphatidylinositol-4,5-bisphosphate 3-kinase activity	23	0.0000
	semaphorin receptor binding	15	0.0000
	protein tyrosine kinase activity	31	0.0000
	tumor necrosis factor-activated receptor activity	15	0.0000
	Ras guanyl-nucleotide exchange factor activity	28	0.0000
	chemorepellent activity	15	0.0000
	semaphorin receptor activity	11	0.0000
	RNA polymerase II transcription factor activity, ligand-activated sequence-specific DNA binding	16	0.0000
	immunoglobulin receptor binding	14	0.0000
	protein binding	471	0.0000
	MHC class II receptor activity	11	0.0000
	heparin binding	30	0.0000
	glycoprotein binding	19	0.0000
	transmembrane signaling receptor activity	34	0.0000
	hormone activity	21	0.0000
	growth factor binding	12	0.0000
	chemoattractant activity	12	0.0000
	transforming growth factor beta receptor binding	14	0.0000
	tumor necrosis factor receptor binding	12	0.0000
	coreceptor activity	12	0.0000
	neuropilin binding	9	0.0000
	protein homodimerization activity	65	0.0000
	MHC class II protein complex binding	9	0.0000
	chemokine receptor activity	9	0.0000
	vascular endothelial growth factor-activated receptor activity	6	0.0001
	transmembrane receptor protein tyrosine kinase activity	11	0.0001
	identical protein binding	60	0.0002
	non-membrane spanning protein tyrosine kinase activity	11	0.0006
	platelet-derived growth factor receptor binding	7	0.0006
	enzyme binding	33	0.0006
	epidermal growth factor receptor binding	9	0.0009
	protein kinase binding	35	0.0011
	beta-2-microglobulin binding	6	0.0012

	receptor tyrosine kinase binding	10	0.0017
	1-phosphatidylinositol-3-kinase activity	10	0.0017
	cytokine binding	7	0.0022
	transforming growth factor beta-activated receptor activity	5	0.0022
	peptide hormone binding	8	0.0023
	virus receptor activity	12	0.0039
	TAP1 binding	4	0.0057
	leukotriene receptor activity	4	0.0057
	retinoid X receptor binding	6	0.0057
	CCR chemokine receptor binding	7	0.0062
	CXCR chemokine receptor binding	5	0.0063
	kinase activity	24	0.0063
	transforming growth factor beta binding	6	0.0074
	receptor signaling protein tyrosine kinase activity	5	0.0098
	signal transducer activity	21	0.0100
	integrin binding	14	0.0101
	vascular endothelial growth factor binding	4	0.0112
	interleukin-20 binding	4	0.0112
	retinoic acid receptor activity	4	0.0112
	CXCR3 chemokine receptor binding	4	0.0112
	G-protein coupled peptide receptor activity	6	0.0119
	steroid binding	7	0.0128
	MHC class I protein binding	6	0.0148
	fatty acid binding	6	0.0148
	protein heterodimerization activity	36	0.0172
	activin binding	5	0.0178
	antioxidant activity	6	0.0183
	T cell receptor binding	4	0.0192
	S100 protein binding	5	0.0237
	type II transforming growth factor beta receptor binding	4	0.0308
	small molecule binding	4	0.0308
	C-X-C chemokine receptor activity	4	0.0308
	transcriptional activator activity, RNA polymerase II core promoter proximal region sequence-specific binding	21	0.0438
	ubiquitin protein ligase binding	24	0.0447
	chemokine receptor binding	4	0.0447
	bile acid binding	4	0.0447
	D-glucose transmembrane transporter activity	4	0.0447
	receptor agonist activity	5	0.0484

Supplementary table 4 The significantly enriched KGEE pathways for 784 FI-DEGs between the normal and patients with KIRC.

Term	Count	FDR
hsa04060:Cytokine-cytokine receptor interaction	113	0.0000
hsa04650:Natural killer cell mediated cytotoxicity	52	0.0000
hsa04612:Antigen processing and presentation	41	0.0000
hsa05323:Rheumatoid arthritis	41	0.0000
hsa05332:Graft-versus-host disease	25	0.0000
hsa05416:Viral myocarditis	32	0.0000
hsa05330:Allograft rejection	26	0.0000
hsa04660:T cell receptor signaling pathway	41	0.0000
hsa04662:B cell receptor signaling pathway	33	0.0000
hsa05166:HTLV-I infection	64	0.0000
hsa04064:NF-kappa B signaling pathway	36	0.0000
hsa05164:Influenza A	51	0.0000
hsa05200:Pathways in cancer	81	0.0000
hsa04672:Intestinal immune network for IgA production	26	0.0000
hsa04062:Chemokine signaling pathway	52	0.0000
hsa05320:Autoimmune thyroid disease	27	0.0000
hsa05162:Measles	43	0.0000
hsa04630:Jak-STAT signaling pathway	45	0.0000
hsa04940:Type I diabetes mellitus	24	0.0000
hsa05168:Herpes simplex infection	49	0.0000
hsa05169:Epstein-Barr virus infection	39	0.0000
hsa05321:Inflammatory bowel disease (IBD)	28	0.0000
hsa04151:PI3K-Akt signaling pathway	70	0.0000
hsa05150:Staphylococcus aureus infection	25	0.0000
hsa04668:TNF signaling pathway	35	0.0000
hsa05145:Toxoplasmosis	35	0.0000
hsa04380:Osteoclast differentiation	38	0.0000
hsa04640:Hematopoietic cell lineage	30	0.0000
hsa05140:Leishmaniasis	27	0.0000
hsa05340:Primary immunodeficiency	19	0.0000
hsa04360:Axon guidance	36	0.0000
hsa04015:Rap1 signaling pathway	48	0.0000
hsa04014:Ras signaling pathway	50	0.0000
hsa05152:Tuberculosis	43	0.0000
hsa04066:HIF-1 signaling pathway	30	0.0000
hsa04514:Cell adhesion molecules (CAMs)	37	0.0000
hsa04620:Toll-like receptor signaling pathway	30	0.0000
hsa05214:Glioma	23	0.0000
hsa04012:ErbB signaling pathway	26	0.0000
hsa04145:Phagosome	35	0.0000
hsa05310:Asthma	15	0.0000
hsa05142:Chagas disease (American trypanosomiasis)	28	0.0000

hsa05218:Melanoma	22	0.0000
hsa04664:Fc epsilon RI signaling pathway	21	0.0000
hsa04210:Apoptosis	20	0.0000
hsa04010:MAPK signaling pathway	45	0.0000
hsa05144:Malaria	17	0.0000
hsa05215:Prostate cancer	23	0.0000
hsa05205:Proteoglycans in cancer	37	0.0000
hsa05161:Hepatitis B	30	0.0000
hsa05160:Hepatitis C	28	0.0000
hsa05211:Renal cell carcinoma	18	0.0000
hsa05134:Legionellosis	16	0.0000
hsa04370:VEGF signaling pathway	17	0.0000
hsa04915:Estrogen signaling pathway	22	0.0001
hsa04510:Focal adhesion	35	0.0001
hsa05230:Central carbon metabolism in cancer	17	0.0001
hsa05220:Chronic myeloid leukemia	18	0.0001
hsa03320:PPAR signaling pathway	17	0.0001
hsa05219:Bladder cancer	13	0.0001
hsa05223:Non-small cell lung cancer	15	0.0002
hsa05231:Choline metabolism in cancer	21	0.0002
hsa04068:FoxO signaling pathway	25	0.0002
hsa05212:Pancreatic cancer	15	0.0010
hsa04670:Leukocyte transendothelial migration	21	0.0013
hsa04666:Fc gamma R-mediated phagocytosis	17	0.0016
hsa04621:NOD-like receptor signaling pathway	13	0.0025
hsa05133:Pertussis	15	0.0040
hsa05120:Epithelial cell signaling in Helicobacter pylori infection	14	0.0040
hsa04722:Neurotrophin signaling pathway	20	0.0051
hsa04920:Adipocytokine signaling pathway	14	0.0059
hsa04080:Neuroactive ligand-receptor interaction	36	0.0064
hsa04917:Prolactin signaling pathway	14	0.0065
hsa05143:African trypanosomiasis	9	0.0067
hsa05221:Acute myeloid leukemia	12	0.0073
hsa04810:Regulation of actin cytoskeleton	29	0.0073
hsa05132:Salmonella infection	15	0.0095
hsa04350:TGF-beta signaling pathway	15	0.0104
hsa04610:Complement and coagulation cascades	13	0.0131
hsa05210:Colorectal cancer	12	0.0152
hsa05322:Systemic lupus erythematosus	20	0.0152
hsa04931:Insulin resistance	17	0.0179
hsa04623:Cytosolic DNA-sensing pathway	12	0.0188
hsa04550:Signaling pathways regulating pluripotency of stem cells	20	0.0231
hsa04932:Non-alcoholic fatty liver disease (NAFLD)	21	0.0249

hsa05146:Amoebiasis	16	0.0315
hsa05213:Endometrial cancer	10	0.0325

Supplementary table 5 The significantly enriched GO term pathways for 226 FI-DEGs between KIRC patients with low risk value and KIRC patients with high risk value.

Category_Term		Count	FDR
BP	immune response	44	0.0000
	complement activation, classical pathway	21	0.0000
	inflammatory response	34	0.0000
	Fc-gamma receptor signaling pathway involved in phagocytosis	21	0.0000
	complement activation	18	0.0000
	response to lipopolysaccharide	22	0.0000
	receptor-mediated endocytosis	22	0.0000
	innate immune response	30	0.0000
	positive regulation of B cell activation	11	0.0000
	defense response to bacterium	19	0.0000
	chemotaxis	17	0.0000
	regulation of immune response	19	0.0000
	Fc-epsilon receptor signaling pathway	19	0.0000
	phagocytosis, recognition	10	0.0000
	B cell receptor signaling pathway	12	0.0000
	cell-cell signaling	21	0.0000
	phagocytosis, engulfment	10	0.0000
	chemokine-mediated signaling pathway	12	0.0000
	positive regulation of cytosolic calcium ion concentration	15	0.0000
	signal transduction	42	0.0000
	cell surface receptor signaling pathway	19	0.0000
	positive regulation of MAPK cascade	11	0.0000
	defense response to virus	14	0.0000
	proteolysis	23	0.0000
	cytokine production	7	0.0000
	positive regulation of cell proliferation	22	0.0000
	retina homeostasis	8	0.0000
	type I interferon signaling pathway	9	0.0001
	cell chemotaxis	9	0.0001
	positive regulation of angiogenesis	11	0.0001
	positive regulation of ERK1 and ERK2 cascade	13	0.0001
	regulation of cell proliferation	13	0.0002
	positive regulation of tumor necrosis factor biosynthetic process	5	0.0004
	positive regulation of NF-kappaB transcription factor activity	11	0.0004
	negative regulation of viral genome replication	7	0.0005

	response to virus	10	0.0005
	positive regulation of interleukin-8 production	6	0.0007
	toll-like receptor signaling pathway	6	0.0009
	negative regulation of apoptotic process	19	0.0010
	astrocyte development	5	0.0016
	positive regulation of chemokine production	5	0.0020
	semaphorin-plexin signaling pathway	6	0.0021
	positive regulation of interferon-beta biosynthetic process	4	0.0024
	positive regulation of leukocyte chemotaxis	5	0.0024
	acute-phase response	6	0.0045
	negative regulation of hormone secretion	4	0.0076
	positive regulation of protein phosphorylation	9	0.0076
	antibacterial humoral response	6	0.0076
	positive regulation of interferon-gamma production	6	0.0092
	regulation of cytokine secretion	4	0.0097
	semaphorin-plexin signaling pathway involved in axon guidance	4	0.0123
	activation of transmembrane receptor protein tyrosine kinase activity	4	0.0123
	positive regulation of nucleotide-binding oligomerization domain containing 2 signaling pathway	3	0.0137
	negative regulation of cation channel activity	3	0.0137
	positive regulation of gene expression	12	0.0164
	positive regulation of peptidyl-tyrosine phosphorylation	7	0.0176
	defense response to Gram-negative bacterium	6	0.0183
	positive regulation of MAP kinase activity	6	0.0248
	positive regulation of interferon-alpha biosynthetic process	3	0.0248
	transmembrane receptor protein tyrosine kinase signaling pathway	7	0.0376
	cytokine-mediated signaling pathway	8	0.0379
	neutrophil chemotaxis	6	0.0386
	positive regulation of Ras protein signal transduction	4	0.0411
	defense response to protozoan	4	0.0411
	negative regulation of cysteine-type endopeptidase activity involved in apoptotic process	6	0.0449
	embryo implantation	5	0.0463
	Ras protein signal transduction	6	0.0464
	interferon-gamma-mediated signaling pathway	6	0.0487
CC	extracellular space	75	0.0000
	extracellular region	81	0.0000
	blood microparticle	20	0.0000
	integral component of plasma membrane	52	0.0000
	external side of plasma membrane	21	0.0000
	plasma membrane	93	0.0000
	immunoglobulin complex, circulating	8	0.0000

	extracellular exosome	61	0.0000
	cell surface	19	0.0017
	endosome membrane	10	0.0067
	receptor complex	8	0.0132
MF	antigen binding	22	0.0000
	serine-type endopeptidase activity	23	0.0000
	growth factor activity	19	0.0000
	immunoglobulin receptor binding	10	0.0000
	chemokine activity	10	0.0000
	cytokine receptor activity	9	0.0000
	receptor binding	20	0.0000
	heparin binding	12	0.0003
	cytokine activity	12	0.0007
	retinoid binding	4	0.0043
	CXCR chemokine receptor binding	4	0.0058
	hormone activity	8	0.0064
	peptide hormone binding	5	0.0107
	steroid hormone receptor activity	6	0.0201
	neuropilin binding	4	0.0217
	transporter activity	10	0.0279
	CXCR3 chemokine receptor binding	3	0.0365
	receptor activity	10	0.0406

Supplementary table 6 The significantly enriched KGEE pathways for 226 FI-DEGs between KIRC patients with low risk value and KIRC patients with high risk value.

Term	Count	FDR
hsa04060:Cytokine-cytokine receptor interaction	32	0.0000
hsa04630:Jak-STAT signaling pathway	17	0.0000
hsa04062:Chemokine signaling pathway	18	0.0000
hsa04620:Toll-like receptor signaling pathway	13	0.0001
hsa05164:Influenza A	13	0.0063
hsa05214:Glioma	8	0.0083
hsa05161:Hepatitis B	11	0.0142
hsa05134:Legionellosis	7	0.0142
hsa04360:Axon guidance	10	0.0184
hsa05219:Bladder cancer	6	0.0208
hsa05162:Measles	10	0.0208
hsa04640:Hematopoietic cell lineage	8	0.0240
hsa04014:Ras signaling pathway	13	0.0249
hsa05200:Pathways in cancer	18	0.0272
hsa04151:PI3K-Akt signaling pathway	16	0.0418

hsa04612:Antigen processing and presentation	7	0.0418
